# Hospital Meetings, &c.

**Published:** 1900-02-10

**Authors:** 


### HOSPITAL MEETINGS, &C.

### LONDON THROAT HOSPITAL.

Dr. Woakes presided at the thirteenth annual meeting of the London Throat Hospital held on Tuesday, 30th ult. The report presented by the committee stated that the new wards opened in 1897 had continued to justify the expenditure on them, 579 in-patients having been admitted for treatment, as against 465 in 1898. The out-patients numbered 3,026, with 15,0[illegible]7 attendances, as compared with 2,814 in 1898, with 13,[illegible]3 attendances. During the year Mr. H. E. Kearley had been elected as chairman to the hospital, in succession to Mr. W. L. Burdett-Coutts. With regret the committee recorded the death of Mr. G. Bailey, whose post as anæsthetist had been filled by Dr. Furniss Potter. Mr. G. C. Wilkin had resigned, and the vacancy on the senior medical staff thus caused had been filled by the appointment of Mr. E. B. Waggett, previously senior assistant surgeon. The matron, Miss Chitty, had resigned her position, which had been filled by Miss Gannon. The financial statement showed ordinary receipts of £[illegible],462, and ordinary expenditure of £1,410, for the past year, a debt of £300 being still left over owing to deficits in former years. The report was adopted, and the retiring committee re-elected. An alteration in the rules was also agreed to, making the quorum of the Executive Committee three instead of four. A vote of thanks to Dr. Waggett was passed, in recognition of special services in connection with an entertainment in aid of the funds. In reply to an inquiry the Secretary stated that the average length of each patient's stay during the year had been 5·2 days. The average cost of each out-patient worked out at 3s. 1d. The proceedings then terminated.

### GENERAL LYING-IN HOSPITAL.

The annual general meeting of the above charity was held on the 31st ult. at the hospital, York Road, Lambeth. Lord Lister presided, and those present included Sir John Williams, Dr. Champneys, Dr. Cullingworth, Mr. Maudsley, Dr. Dakin, and Dr. Boxall. Sympathetic reference was made to the death of the vice-chairman, Mr. J. Eardley Hill, and it was resolved to send Mrs. Hill a vote of condolence from the committee. It was also stated that a bronze tablet was to be placed in one of the wards of the hospital as a memorial of the amount of good work for the General Lying-in Hospital which Mr. Hill had done, and of the loss which the hospital consequently sustains in his death.

The report for 1899 showed that 715 letters had been issued to in-patients and 1,706 to out-patients, the number of in-patients admitted showing an increase of fourteen over the previous year, although the accommodation available was the same. The convalescent home at Woking established through the generosity of Mrs. Johnstone, which was opened on October 31st last, had already proved of great advantage, and during the two months from the date of its opening to the end of the year six mothers and their infants were admitted to it. The expenditure for the year was £3,409 18s. 7d., the income being £3,362 19s. 7d., of which £1,347 9s. were the fees received for training nurses, 82 of whom passed through the school during the year and 26 midwives, all receiving certificates. The medical report showed that of the 560 patients who were admitted only three had died, and no septic illness had arisen in the hospital during the past year. The number of patients attended at their own homes, viz., 1,667, was less than the previous year, in consequence of the curtailment of the district assigned to the midwives, which curtailment had proved advantageous to the hospital. In moving the adoption of the report, Dr. Boxall brought forward some interesting particulars showing the work of the hospital during the last 21 years, when it was commenced to be conducted on aseptic lines. During that period there had been 8,618 deliveries and 43 deaths, 13 of which occurred from septicæmia, 18 from accidents of childbirth, and 12 from other causes. It was pointed out that no death from sepsis arising in the hospital had occurred during the past 11 years, during which time there had been 5,715 deliveries; three cases had however, been sent in already infected, but the disease had not spread to any other case, which showed the adequate aseptic precautions which were taken. Sir John Williams moved that Lord Lister be re-elected president for the ensuing year, which motion was carried with expression of great approval, and a vote of thanks to Lord Lister for presiding was also heartily carried. The medical officers, committee of management, and auditors were also re-elected and thanked for their services during the past year.

